# Clinical Relevance of Calcium Measures: QT-Based Comparison of Ionized, Total, and Albumin-Corrected Calcium

**DOI:** 10.34067/KID.0000001236

**Published:** 2026-05-26

**Authors:** Hiroaki Miyauchi, Tatsuhiko Azegami, Takashin Nakayama, Kaori Hayashi

**Affiliations:** Division of Nephrology, Endocrinology, and Metabolism, Department of Internal Medicine, Keio University School of Medicine, Tokyo, Japan

**Keywords:** calcium, electrolytes

## Abstract

**Key Points:**

- Ionized calcium showed the strongest associations with the QT interval, whereas total and corrected calcium showed weaker associations.
- Calcium measures that correlated better with ionized calcium did not necessarily perform better for QT-based outcomes.
- These findings reinforced ionized calcium as the most clinically valid reference and supported outcome-based calcium assessment.

**Background:**

Ionized calcium (iCa) is the physiologically active fraction of circulating calcium, whereas total calcium (tCa) and albumin-corrected calcium (cCa) remain widely used. Despite growing concerns over the accuracy of correction formulas, most evaluations rely on concordance with iCa rather than meaningful physiologic outcomes. We compared iCa, tCa, and cCa and assessed their clinical validity using ventricular repolarization—reflected by the QT interval—as a physiologic end point.

**Methods:**

This single-center cross-sectional observational study included adults who visited our outpatient department with simultaneous measurements of iCa, tCa, electrolytes, albumin, and electrocardiography. iCa, tCa, and seven albumin-correction formulas were examined. Associations with the QT interval were evaluated using linear regression and restricted cubic splines; logistic regression assessed corrected QT prolongation. Sensitivity analyses included alternative QT-correction methods and analyses in hypoalbuminemia.

**Results:**

Among 647 participants (median age 68 years; 54% male), common comorbidities included hypertension (33%), diabetes (18%), and cardiac disease (11%). Correlations between iCa and other calcium measures varied widely. In fully adjusted analyses, iCa demonstrated the strongest association with QT interval (standardized *β*=−0.22; 95% confidence interval, −0.29 to −0.14). tCa and all cCa exhibited substantially weaker associations. Although some tCa and cCa demonstrated relatively higher correlation with iCa, they exhibited weaker associations with QT. In hypoalbuminemia (albumin <3.0 g/dl, *n*=118), agreement with iCa declined for all cCa, and associations with QT were attenuated; only iCa (standardized *β*=−0.25; 95% confidence interval, −0.44 to −0.06) and limited formulas (notably Payne) retained modest associations.

**Conclusions:**

iCa is the physiologically and clinically valid measure of calcium status consistently demonstrating the strongest associations with ventricular repolarization. By contrast, tCa and cCa demonstrate substantially reduced clinical relevance and numerical concordance with iCa does not reflect clinical validity. These findings support outcome-based validation of calcium measure and reinforce the importance of direct iCa measurement.

## Visual Abstract

**Figure:**
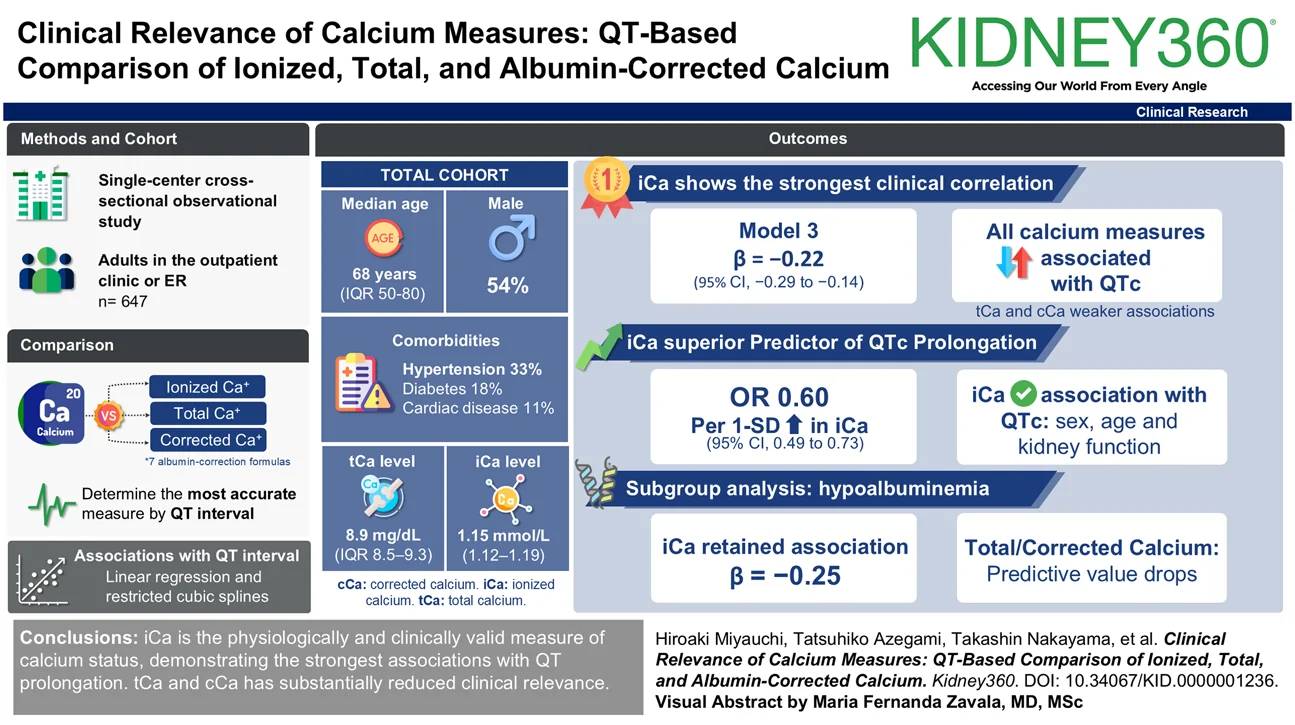

## Introduction

Serum calcium circulates in several forms, including ionized and protein-bound fractions, with approximately half present as physiologically active ionized calcium (iCa).^1^ To account for the effect of protein binding, formulas that correct total calcium (tCa) for serum albumin have been widely used for decades.^2,3^ However, accumulating evidence suggests that albumin-corrected calcium (cCa) does not consistently align with iCa,^4^ raising concerns that these correction equations may diminish, rather than enhance, diagnostic accuracy. Despite renewed scrutiny of these long-standing correction formulas, there remains a paucity of evidence evaluating their performance against clinically meaningful physiologic outcomes.

Calcium plays a central role in myocardial excitation-contraction coupling, and alterations in calcium levels affect the QT interval on electrocardiography (ECG).^5–8^ QT prolongation is a well-established predictor of cardiovascular disease, malignant arrhythmias, and sudden cardiac death. Moreover, epidemiologic studies have demonstrated that variations in QT-interval duration—even within conventional reference ranges—are associated with higher mortality risk, indicating that QT duration represents a clinically relevant marker of calcium-related electrophysiologic effects.^9–13^ Accordingly, the QT interval serves as a meaningful surrogate for calcium-related physiologic perturbations.

Given these considerations, this study sought to characterize the interrelationships of tCa and cCa with iCa and to determine which measure most accurately reflects calcium physiology by examining their associations with the QT interval on ECG. In contrast to prior work that has primarily compared calcium measures based on concordance with iCa alone, our approach leverages a clinically relevant outcome to provide a more robust assessment of their clinical utility.

## Methods

### Study Population

This single-center cross-sectional observational study was conducted at Keio University Hospital. We included adult patients (≥18 years) who visited our outpatient department—including emergency or unscheduled visits—between January 2012 and June 2025 and had simultaneous measurements of iCa, tCa, albumin (Alb), potassium (K), phosphorus (P), magnesium (Mg), and ECG. ECGs were required to be performed within 2 hours of blood sampling and before any therapeutic intervention. Exclusion criteria were: (*1*) cardiac arrest at presentation; (*2*) prior pacemaker implantation; (*3*) atrial fibrillation on the presenting ECG; (*4*) second- or third-degree atrioventricular block on the presenting ECG; (*5*) QRS duration >120 ms; and (*6*) a diagnosis of acute coronary syndrome at presentation.^8,14^ All consecutive eligible patients during the study period were included. Participants were included regardless of calcium status, and analyses therefore covered the full spectrum of hypocalcemia, normocalcemia, and hypercalcemia.

The study was approved by the Ethics Committee of the Keio University School of Medicine (approval number: 2025-1188) and conducted in accordance with the principles of the Declaration of Helsinki. Because the study used pre-existing clinical records, informed consent was obtained through an opt-out process.

### Data Collection

Clinical data were obtained from electronic medical records. Collected variables included age, sex, ECG findings, systolic and diastolic BP, serum creatinine (Cr), eGFR, Alb, K, P, Mg, and potential hydrogen (pH). Medication data were collected at the pharmacologic class level and included the use of QT-prolonging medications, antihypertensive agents, and diuretics. Extracted comorbidities were diabetes mellitus, hypertension, cardiac disease (myocardial infarction, heart failure, or valvular disease), and malignancy. eGFR was calculated using the equation recommended by the Japanese Society of Nephrology.^15^

$$\text{eGFR}\;\left(\text{ml}/\min\;\text{per}\;1.73\;m^{2}\right)=194\times {\text{Cr}}^{-1.094}\times {\text{age}}^{-0.287}\;\left(\times 0.739\;\text{for}\;\text{females}\right)$$

QT-prolonging medications were identified according to the CredibleMeds risk categories (known, possible, or conditional) defined by the Arizona Center for Education and Research on Therapeutics (Supplemental Table 1).^16^ Serum Alb, Cr, K, Ca, P, and Mg were measured using automated chemistry analyzers (Labospect 008 or Labospect 008*α*; Hitachi High-Tech, Tokyo, Japan). iCa and pH were measured using whole blood samples on a blood gas analyzer (ABL90 FLEX or ABL90 FLEX PLUS; Radiometer Medical, Copenhagen, Denmark). cCa was calculated using seven albumin-correction formulas and used for analyses (Supplemental Table 2).^2,3,17–21^

### ECG Analysis

Resting standard 12-lead ECGs were recorded using automated analysis systems (Nihon Kohden ECG-1450, ECG-1550, or ECG-2450). QT and RR intervals were obtained from device-generated automated measurements and recorded in milliseconds. The corrected QT (QTc) interval was calculated using Bazett formula (QTc=QT/√RR).^22^ QT prolongation was defined as QTc >450 ms in men and >460 ms in women.^23^

### Statistical Analysis

Baseline characteristics are presented as medians with interquartile ranges for continuous variables and as counts with percentages for categorical variables. Relationships between iCa and either tCa or cCa were assessed using Pearson correlation coefficients, and fitted regression lines were displayed in scatterplots. These analyses were descriptive and intended to illustrate the variability in numerical concordance across formulas; formal statistical comparisons between correlation coefficients were not performed. We next evaluated categorical agreement with iCa, categorized as hypocalcemia (<1.15 mmol/L), normocalcemia (1.15–1.35 mmol/L), or hypercalcemia (>1.35 mmol/L). tCa and each cCa measure were categorized using corresponding cutoffs (<8.4, 8.4–10.2, and >10.2 mg/dl). Agreement was quantified using percent agreement and weighted kappa statistics (κ).

For the primary analysis, linear regression models were constructed with QTc as the dependent variable and each calcium measure (iCa, tCa, or cCa) as the independent variable. Model 1 was adjusted for age and sex; model 2 was additionally adjusted for cardiac disease and the use of QT-prolonging medications; and model 3 further included eGFR, K, P, Mg, hypertension, and diabetes. Results are presented as standardized regression coefficients (*β*) with 95% confidence intervals (CIs). Because multiple calcium measures were compared, we applied a false discovery rate correction using the Benjamini-Hochberg procedure across the *P* values corresponding to the associations of each calcium measure with the outcome. To evaluate potential nonlinear associations between QTc and each calcium measure, restricted cubic spline models were fitted with knots placed at the 5th, 35th, 65th, and 95th percentiles.

In secondary analyses, logistic regression was performed with QTc prolongation as the dependent variable, and odds ratios (ORs) with 95% CIs were calculated. To enable direct comparison across iCa, tCa, and cCa, all calcium measures were standardized and associations were expressed per 1-SD increase. The same stepwise adjustment strategy used in the primary analysis was applied. In subgroup analyses, potential effect modification was evaluated by sex (male versus female), age (<65 versus ≥65 years), and kidney function (eGFR <60 versus ≥60 ml/min per 1.73 m^2^), using the fully adjusted model (model 3).

To assess the robustness of our findings, the following sensitivity analyses were performed: (*1*) repeating the primary analysis using uncorrected QT as the outcome while adjusting for RR as an additional covariate; (*2*) recalculating QTc using the Hodges formula^24^; (*3*) restricting the analysis to participants with hypoalbuminemia (Alb <3.0 g/dl); and (*4*) stratifying participants according to the presence or absence of alkalemia based on blood gas pH values. Although Bazett formula is the most widely used QT-correction method, it has been criticized for overestimating QT prolongation. Therefore, we included as sensitivity analyses both the RR-adjusted approach employed in previous large-scale studies^8,14^ and the Hodges formula, which has been associated with mortality risk related to QTc.^25^

All statistical analyses were performed using IBM SPSS Statistics version 30 and R version 4.5.1.

## Results

### Characteristics of the Study Population

A total of 647 participants were included in the analysis (Figure 1). The median age was 68 (interquartile range, 50–80) years, and 54% were male (Table 1). In total, 177 participants (27%) were taking at least one QT-prolonging medication (Supplemental Table 1). Common comorbidities included hypertension (33%), diabetes (18%), cardiac disease (11%), and malignancy (8%), indicating that the cohort represents a clinically heterogeneous population rather than healthy individuals. The median tCa level was 8.9 (8.5–9.3) mg/dl, and the median iCa level was 1.15 (1.12–1.19) mmol/L. The median RR and QT intervals were 690 (588–822) and 376 (348–406) ms, respectively. QTc prolongation was observed in 267 participants (41%).

**Figure 1:**
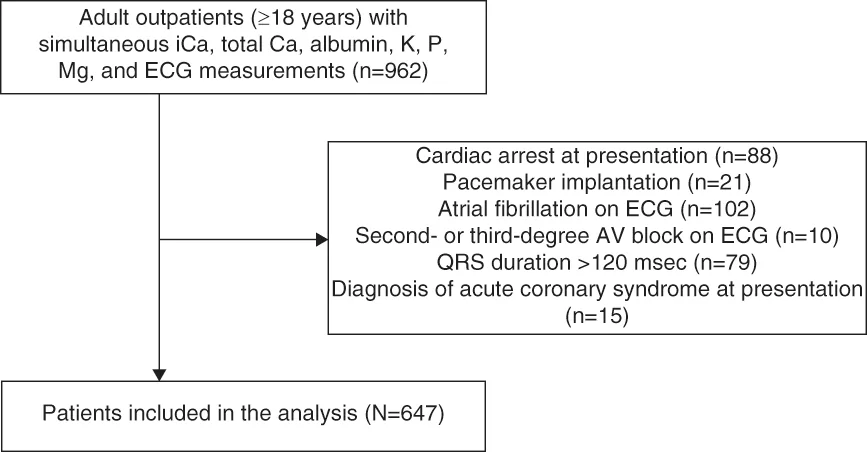
**Patient selection flow chart.** AV block, atrioventricular block; Ca, calcium; ECG, electrocardiography; iCa, ionized calcium; K, potassium; Mg, magnesium; P, phosphorus.

**Table 1:** Baseline characteristics

Variables	Overall (*N*=647)
Age, yr	68 (50–80)
Male, *n* (%)	351 (54)
**BP, mm Hg**	
sBP	130 (110–154)
dBP	80 (67–95)
**Comorbidities, *n* (%)**	
Diabetes	118 (18)
Hypertension	211 (33)
Cardiac disease	74 (11)
Malignancy	50 (8)
**Medications, *n* (%)**	
RAS inhibitors	107 (17)
*β* blockers	59 (9)
Thiazide diuretics	9 (1)
Loop diuretics	63 (10)
QT-prolonging medications	177 (27)
**Laboratory values**	
Potassium, mEq/L	4.1 (3.7–4.5)
tCa, mg/dl	8.9 (8.5–9.3)
iCa, mmol/L	1.15 (1.12–1.19)
Phosphate, mg/dl	3.3 (2.7–4.0)
Magnesium, mg/dl	2.1 (1.9–2.2)
Albumin, g/dl	3.8 (3.1–4.2)
Cr, mg/dl	0.84 (0.65–1.20)
eGFR, ml/min per 1.73 m^2^	64 (41–84)
pH	7.42 (7.37–7.46)
**ECG**	
RR interval, ms	690 (588–822)
QT interval, ms	376 (348–406)
QTc interval, ms	449 (430–470)
QTc prolongation, *n* (%)	267 (41)

Values are expressed as median (interquartile range) or number (percentage). Cr, creatinine; dBP, diastolic BP; ECG, electrocardiography; iCa, ionized calcium; pH, potential hydrogen; QTc, corrected QT; RAS, renin-angiotensin system; sBP, systolic BP; tCa, total calcium.

### Association between iCa and Other Calcium Measures

Correlations between iCa and tCa or each cCa varied widely (Table 2 and Supplemental Figure 1). The strongest associations with iCa were observed for the Antonio formula (R^2^=0.57), followed by the James formula (R^2^=0.53) and tCa (R^2^=0.52). By contrast, several formulas—including the Simplified and Berry formulas—showed substantially weaker associations with iCa (R^2^=0.34 and 0.27, respectively). Agreement with iCa categories (hypocalcemia/normocalcemia/hypercalcemia) also varied across formulas (Table 2 and Supplemental Table 3). The highest agreement was observed for tCa (70%; κ=0.38 [95% CI, 0.31 to 0.45]), the Orrell formula (63%; κ=0.23 [95% CI, 0.16 to 0.30]), the Antonio formula (63%; κ=0.20 [95% CI, 0.15 to 0.26]), and the James formula (63%; κ=0.20 [95% CI, 0.15 to 0.26]). Taken together, tCa, as well as the Antonio and James formulas, demonstrated comparatively better performance in terms of both correlation with and categorical agreement to iCa.

**Table 2:** Association (R^2^) with ionized calcium, classification agreement, and weighted κ

Calcium Measure	R^2^	Classification Agreement, %	κ (95% CI)
tCa	0.52	70	0.38 (0.31 to 0.45)
Payne formula	0.30	56	0.13 (0.09 to 0.18)
Simplified formula	0.34	58	0.13 (0.08 to 0.18)
James formula	0.53	63	0.20 (0.15 to 0.26)
Orrell formula	0.41	63	0.23 (0.16 to 0.30)
Berry formula	0.27	52	0.12 (0.08 to 0.17)
Thode formula	0.32	56	0.15 (0.10 to 0.19)
Antonio formula	0.57	63	0.20 (0.15 to 0.26)

CI, confidence interval; tCa, total calcium.

### Primary Outcomes

Restricted cubic spline analyses showed linear inverse associations between each calcium measure and QT interval, with no evidence of threshold effects (Figure 2). In linear regression analyses, iCa consistently showed the strongest association with QTc. In model 3, the standardized regression coefficient for iCa was *β*=−0.22 (95% CI, −0.29 to −0.14; Table 3). Although all calcium measures were inversely associated with QTc, the effect sizes for tCa and the various cCa were consistently smaller than those for iCa, and differences among the correction formulas were minimal (Table 3).

**Figure 2:**
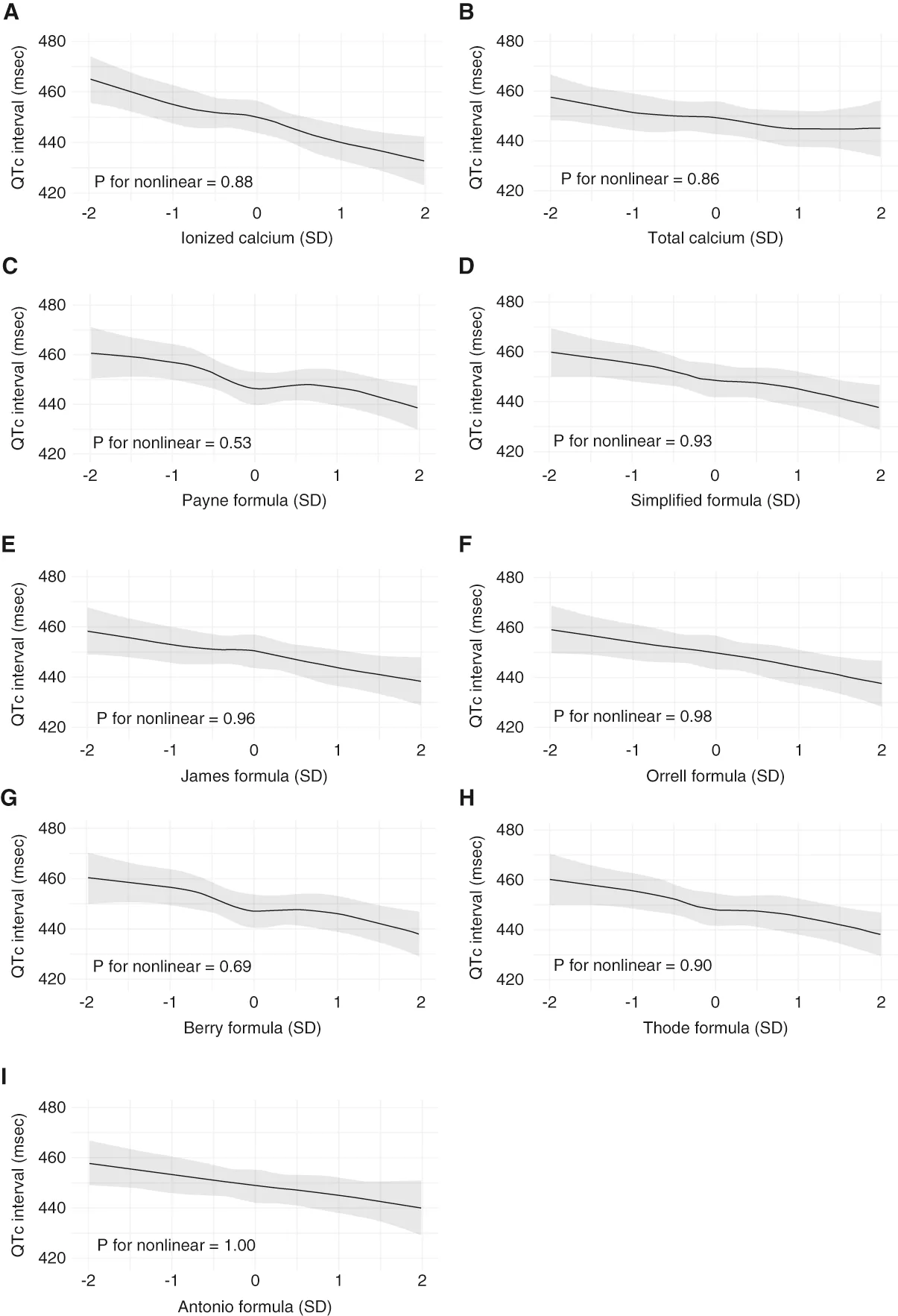
**Restricted cubic spline curves for the association between QTc interval and calcium measures.** Restricted cubic spline models (knots at the 5th, 35th, 65th, and 95th percentiles) were fitted using model 3 to assess the associations between QTc interval and (A) iCa, (B) tCa, (C) Payne formula, (D) simplified formula, (E) James formula, (F) Orrell formula, (G) Berry formula, (H) Thode formula, and (I) Antonio formula. Solid lines represent spline estimates, shaded areas indicate 95% CIs, and *P* values for nonlinearity are shown in each panel. One SD corresponded to 0.073 mmol/L for iCa, 0.706 mg/dl for tCa, 0.623 mg/dl for Payne, 0.563 mg/dl for simplified, 0.547 mg/dl for James and Orrell, 0.589 mg/dl for Berry, 0.617 mg/dl for Thode, and 0.041 mmol/L for Antonio formulas. CI, confidence interval; QTc, corrected QT; tCa, total calcium.

**Table 3:** Association between corrected QT interval and ionized, total, and corrected calcium

Calcium Measure	Standardized *β* (95% CI)				
	Unadjusted	Model 1	Model 2	Model 3	FDR-Adjusted *P* Value (Model 3)
iCa	−0.24 (−0.31 to −0.16)	−0.24 (−0.31 to −0.16)	−0.23 (−0.31 to −0.16)	−0.22 (−0.29 to −0.14)	<0.001
tCa	−0.13 (−0.20 to −0.05)	−0.12 (−0.20 to −0.04)	−0.11 (−0.19 to −0.03)	−0.09 (−0.17 to −0.01)	0.02
**cCa**					
Payne formula	−0.09 (−0.17 to −0.02)	−0.14 (−0.22 to −0.06)	−0.15 (−0.23 to −0.07)	−0.14 (−0.22 to −0.06)	0.001
Simplified formula	−0.11 (−0.19 to −0.04)	−0.15 (−0.22 to −0.07)	−0.15 (−0.23 to −0.08)	−0.14 (−0.22 to −0.06)	0.001
James formula	−0.14 (−0.21 to −0.06)	−0.14 (−0.22 to −0.07)	−0.14 (−0.22 to −0.07)	−0.13 (−0.20 to −0.05)	0.002
Orrell formula	−0.12 (−0.20 to −0.05)	−0.15 (−0.22 to −0.07)	−0.15 (−0.23 to −0.07)	−0.14 (−0.21 to −0.06)	0.001
Berry formula	−0.10 (−0.18 to −0.03)	−0.14 (−0.22 to −0.06)	−0.15 (−0.23 to −0.07)	−0.14 (−0.22 to −0.06)	0.001
Thode formula	−0.11 (−0.19 to −0.04)	−0.14 (−0.22 to −0.07)	−0.15 (−0.23 to −0.07)	−0.14 (−0.22 to −0.07)	0.001
Antonio formula	−0.14 (−0.21 to −0.06)	−0.14 (−0.21 to −0.06)	−0.13 (−0.21 to −0.06)	−0.11 (−0.19 to −0.04)	0.005

Model 1 was adjusted for age and sex. Model 2 was adjusted for model 1 covariates, cardiac disease, and QT—prolonging medications. Model 3 was adjusted for model 2 covariates, eGFR, serum potassium, phosphate, magnesium levels, hypertension, and diabetes. *P* values were adjusted for multiple comparisons across calcium measures in model 3 using the Benjamini-Hochberg false discovery rate method. *β*, regression coefficient; cCa, corrected calcium; CI, confidence interval; FDR, false discovery rate; iCa, ionized calcium; tCa, total calcium.

### Secondary Outcomes

QTc prolongation was observed in 267 participants (41%). In logistic regression analyses with QTc prolongation as the outcome, iCa consistently demonstrated the strongest association (Table 4). In model 3, the OR for QTc prolongation per 1-SD increase in iCa was 0.60 (95% CI, 0.49 to 0.73). Although all calcium measurements were significantly associated with QTc prolongation, the magnitudes of these associations were similar across the different correction formulas and were generally weaker than those observed for iCa (Table 4).

**Table 4:** Association between corrected QT prolongation and ionized, total, and corrected calcium

Calcium Measure	OR (95% CI)				
	Unadjusted	Model 1	Model 2	Model 3	FDR-Adjusted *P* Value (Model 3)
iCa	0.58 (0.48 to 0.79)	0.59 (0.48 to 0.71)	0.59 (0.49 to 0.72)	0.60 (0.49 to 0.73)	<0.001
tCa	0.78 (0.66 to 0.92)	0.79 (0.67 to 0.93)	0.80 (0.68 to 0.94)	0.83 (0.70 to 0.99)	0.04
**cCa**					
Payne formula	0.78 (0.66 to 0.92)	0.73 (0.61 to 0.87)	0.71 (0.60 to 0.86)	0.73 (0.60 to 0.88)	0.002
Simplified formula	0.75 (0.63 to 0.89)	0.72 (0.60 to 0.86)	0.71 (0.59 to 0.85)	0.73 (0.60 to 0.87)	0.001
James formula	0.73 (0.61 to 0.87)	0.73 (0.61 to 0.87)	0.73 (0.62 to 0.87)	0.76 (0.63 to 0.90)	0.003
Orrell formula	0.73 (0.62 to 0.87)	0.72 (0.60 to 0.86)	0.71 (0.59 to 0.85)	0.73 (0.61 to 0.88)	0.001
Berry formula	0.76 (0.64 to 0.90)	0.72 (0.61 to 0.86)	0.71 (0.59 to 0.85)	0.73 (0.60 to 0.87)	0.001
Thode formula	0.75 (0.63 to 0.89)	0.72 (0.60 to 0.86)	0.71 (0.59 to 0.85)	0.73 (0.60 to 0.87)	0.001
Antonio formula	0.74 (0.63 to 0.88)	0.75 (0.64 to 0.89)	0.76 (0.64 to 0.90)	0.79 (0.66 to 0.93)	0.008

Odds ratio is expressed per 1-SD increase in each calcium measure.

Model 1 was adjusted for age and sex. Model 2 was adjusted for model 1 covariates, cardiac disease, and QT—prolonging medications. Model 3 was adjusted for model 2 covariates, eGFR, serum potassium, phosphate, magnesium levels, hypertension, and diabetes. *P* values were adjusted for multiple comparisons across calcium measures in model 3 using the Benjamini-Hochberg false discovery rate method. cCa, corrected calcium; CI, confidence interval; FDR, false discovery rate; iCa, ionized calcium; OR, odds ratio; tCa, total calcium.

### Subgroup Analyses

In subgroup analyses, iCa consistently demonstrated the strongest association with QTc across all strata, including sex, age (<65 versus ≥65 years), and kidney function (eGFR <60 versus ≥60 ml/min per 1.73 m^2^). Tests for interaction revealed no significant effect modification for either the standardized *β* coefficients or the ORs for QTc prolongation, indicating that the direction and magnitude of the associations were largely consistent across these clinically relevant subgroups (Supplemental Figure 2).

### Sensitivity Analyses

In sensitivity analyses, models using uncorrected QT as the dependent variable with additional adjustment for RR interval, as well as analyses employing QTc calculated using the Hodges formula, yielded results that were consistent with those obtained in the primary and secondary analyses based on Bazett formula (Supplemental Tables 4–6). In analyses restricted to participants with hypoalbuminemia (*n*=118), associations (R^2^) with iCa ranged from 0.48 to 0.54, and categorical agreement was highest for tCa (75%; κ=0.42 [95% CI, 0.24 to 0.59]; Supplemental Table 7). In linear regression analyses, iCa remained significantly associated with QTc (standardized *β*=−0.25; 95% CI, −0.44 to −0.06), whereas associations for all other calcium measures were no longer significant. In logistic regression analyses, only a few correction formulas—including the Payne formula—retained weak associations with QTc prolongation (Supplemental Tables 8 and 9). Furthermore, stratification by alkalemia status showed a similar overall pattern (Supplemental Tables 10 and 11). iCa remained significantly associated with QTc and QTc prolongation in both strata, whereas tCa was NS after adjustment. Several cCa formulas showed weaker associations, and some lost significance in participants with pH >7.45.

## Discussion

In this study of 647 participants, we found that iCa showed the strongest association with both the QT interval and QT prolongation. These findings reinforce that direct measurement of iCa most accurately reflects physiologically relevant calcium status. Although numerous correction formulas have been developed to improve the numerical agreement between tCa and iCa, our findings indicate that neither tCa nor any cCa performed better than iCa in reflecting physiologically meaningful calcium status. Moreover, the contributions of tCa and cCa to QT prolongation were independent of their numerical correlation with iCa. This observation provides an important perspective for reconsidering the clinical utility and limitations of tCa and cCa.

The QT interval reflects ventricular repolarization and is an established marker of risk for cardiovascular disease, malignant arrhythmias, and sudden cardiac death.^9–12^ Because calcium plays a central role in phase 2 (plateau phase) of the cardiac action potential, alterations in calcium levels directly influence repolarization time.^26^ The QT interval is therefore a meaningful physiologic outcome through which the clinical relevance of calcium status can be evaluated. Large population-based studies such as the Third National Health and Nutrition Examination Survey and the Atherosclerosis Risk in Communities study have demonstrated inverse associations of both iCa and tCa with the QT interval, with consistently stronger associations observed for iCa,^14^ a pattern reproduced in our study. These findings collectively underscore that iCa, which represents the physiologically active, unbound fraction of circulating calcium, most accurately reflects calcium's biologic effects relevant to myocardial repolarization. By contrast, tCa and cCa are influenced by factors such as albumin concentration, pH, and other nonphysiologic determinants. Although these measures may correlate numerically with iCa, they do not fully capture the physiologically active fraction, which likely explains their weaker associations with the QT interval. Our findings reinforce that iCa is the most clinically valid metric for assessing calcium status in contexts where physiologic function is of primary importance.

Most previous studies have focused on evaluating correction formulas based solely on their numerical performance—primarily their correlation with iCa and their diagnostic agreement in classifying hypocalcemia and hypercalcemia.^4,27–29^ For example, a large-scale study of over 22,000 participants compared correlations and categorical agreement between iCa and either tCa or several cCa, concluding that tCa may outperform cCa, particularly in individuals with hypoalbuminemia.^4^ However, these assessments were limited to how well each measure matched iCa or reproduced calcium categories, without examining clinically meaningful outcomes such as QT prolongation.

In recent years, active measurement of iCa has been increasingly recommended in specific clinical settings.^30^ Nevertheless, several guidelines for hyperparathyroidism continue to endorse the widely used albumin-correction formula.^31,32^ Although direct measurement of iCa is unequivocally the most accurate approach, its measurement requires strict preanalytical conditions and is not universally available in routine clinical practice. As a result, tCa and albumin-cCa are still commonly used as practical substitutes in many health care settings.^30^ Within these practical limitations, it is noteworthy that some traditional correction formulas retained limited but detectable associations with QT-related outcomes. Nevertheless, it should also be emphasized that all correction formulas consistently underperformed when compared with iCa.

A strength of this study is that it is the first to evaluate multiple calcium correction formulas using a clinically meaningful outcome—the QT interval on ECG—rather than relying solely on correlation analyses. By directly comparing iCa, tCa, and various cCa within the same population, we were able to clearly demonstrate the discrepancy in clinical validity between iCa and the other calcium measures. Moreover, the consistency of our findings across extensive sensitivity analyses—including alternative QT correction methods and analyses limited to those with hypoalbuminemia—further supports the robustness of our results.

This study has several limitations. First, this was a single-center retrospective study, and selection bias as well as measurement-related influences cannot be fully excluded. However, the use of standardized procedures and the same equipment within a single institution likely minimized variability in the measurements. Second, although major known confounders were adjusted for in multivariable analyses, residual confounding from unmeasured factors such as body habitus cannot be entirely ruled out. Third, because this was a cross-sectional analysis, we were unable to evaluate prognostic outcomes such as incident arrhythmias, hospitalization, or mortality, which should be addressed in future studies. Fourth, although calcium fluctuations can manifest in a broad spectrum of clinical consequences, such as neurologic disturbances and kidney dysfunction, these outcomes were not evaluated. In addition, the QT interval, used as a surrogate of calcium-related cardiac electrophysiology, can be influenced by multiple factors beyond calcium, including heart rate, medications, comorbidities, and other electrolyte disturbances. Although we adjusted for major confounders in multivariable analyses, residual confounding cannot be completely excluded. Therefore, QTc may not fully capture all biologic effects of calcium imbalance. Fifth, our cohort consisted of patients presenting to the emergency department or for unscheduled outpatient visits, which may limit the generalizability of the findings to the broader population. In addition, because the cohort included patients with diverse clinical backgrounds—including single-visit consultations and acute presentations—the underlying causes of hypoalbuminemia were not systematically evaluated, which may affect the generalizability of the findings in hypoalbuminemic patients.

In conclusion, this study demonstrates that iCa is the physiologically and clinically relevant measure of calcium status, consistently showing the strongest association with QT interval and QT prolongation. By contrast, tCa and cCa exhibit weaker and less reliable associations, and even correction formulas that improve numerical agreement with iCa do not necessarily translate into greater clinical relevance. Our findings highlight the value of outcome-based evaluation of calcium measures and suggest that iCa more closely reflects physiologically relevant calcium activity than total or albumin-cCa.

## Supplementary Material

## Data Availability

Original data generated for the study will be made available upon reasonable request to the corresponding author. Data Type: Observational Data and Research Protocols. Reason for Restricted Access: The data underlying this study cannot be made fully publicly available due to institutional policies and ethical considerations related to patient confidentiality. Although the dataset has been de-identified, it was originally collected from clinical records, and there remains a potential risk of re-identification. However, de-identified data and the study protocol can be made available from the corresponding author upon reasonable request, subject to approval by the Institutional Review Board and data use agreements, where applicable.

## Declarative Statements

This study includes clinical experimentation and received Institutional Review Board or Ethics Committee approval. The need to obtain informed patient consent was waived. This study includes clinical experimentation and complies with the Declaration of Helsinki.

## Supplemental Material

Supplemental Table 1. List of medications associated with QT prolongation.

Supplemental Table 2. Calcium correction formulas.

Supplemental Table 3. Cross-classification between calcium measures and iCa categories.

Supplemental Table 4. Association between QT interval and iCa, tCa, and cCa (RR interval additionally adjusted).

Supplemental Table 5. Association between QTc interval (Hodges formula) and iCa, tCa, and cCa.

Supplemental Table 6. Association between QTc prolongation (Hodges formula) and iCa, tCa, and cCa.

Supplemental Table 7. Association (R^2^) with iCa, classification agreement and weighted κ with hypoalbuminemia.

Supplemental Table 8. Association between QTc interval and iCa, tCa, and cCa in individuals with hypoalbuminemia.

Supplemental Table 9. Association between QTc prolongation and iCa, tCa, and cCa in individuals with hypoalbuminemia.

Supplemental Table 10. Association between QTc interval and iCa, tCa, and cCa in individuals stratified by alkalemia status.

Supplemental Table 11. Association between QTc prolongation and iCa, tCa, and cCa in individuals stratified by alkalemia status.

Supplemental Figure 1. Scatter plot and correlation with iCa.

Supplemental Figure 2. Associations of calcium indices with QTc interval and QTc prolongation across clinical subgroups.
