# Supplementary material for: Clinical Relevance of Calcium Measures: QT-Based Comparison of Ionized, Total, and Albumin-Corrected Calcium
Source: Kidney360. 2026 May 26;7(9):1998–2007. doi: 10.34067/KID.0000001236 (PMC13641536; doi:10.34067/KID.0000001236)

Supplemental Material

Supplemental Table 1. List of medications associated with QT prolongation
Supplemental Table 2. Calcium correction formulas
Supplemental Table 3. Cross-classification between calcium measures and ionized calcium categories
Supplemental Table 4. Association between QT interval and ionized, total, and corrected calcium (RR interval additionally adjusted)
Supplemental Table 5. Association between QTc interval (Hodges formula) and ionized, total, and corrected calcium
Supplemental Table 6. Association between QTc prolongation (Hodges formula) and ionized, total, and corrected calcium
Supplemental Table 7. Association (R^2) with ionized calcium, classification agreement and weighted κ with hypoalbuminemia
Supplemental Table 8. Association between QTc interval and ionized, total, and corrected calcium in individuals with hypoalbuminemia
Supplemental Table 9. Association between QTc prolongation and ionized, total, and corrected calcium in individuals with hypoalbuminemia
Supplemental Table 10. Association between QTc interval and ionized, total, and corrected calcium in individuals stratified by alkalemia status
Supplemental Table 11. Association between QTc prolongation and ionized, total, and corrected calcium in individuals stratified by alkalemia status
Supplemental Figure 1. Scatter plot and correlation with ionized calcium
Supplemental Figure 2. Associations of calcium indices with QTc interval and QTc prolongation across clinical subgroups

Supplemental Table 1. List of medications associated with QT prolongation

QT-prolonging medications	n	QT-prolonging medications	n
Amiodarone	5	Lansoprazole	36
Aripiprazole	3	Levofloxacin	5
Atomoxetine	1	Levomepromazine	1
Cilostazol	10	Loperamide	2
Clarithromycin	3	Metoclopramide	6
Clomipramine	1	Mirabegron	1
Disopyramide	2	Mirtazapine	1
Donepezil	7	Omeprazole	4
Eperisone	3	Olanzapine	2
Erythromycin	2	Quetiapine	8
Esomeprazole	38	Risperidone	5
Furosemide	57	Sertraline	2
Galantamine	2	Sulpiride	2
Hydroxyzine	1	Tacrolimus	5
Itraconazole	1	Tramadol	5
Ivabradine	1	Trazodone	4

The listed medications are classified as known or suspected to have QT-prolonging potential according to CredibleMeds® risk categories.

The numbers indicate prescription counts.

Supplemental Table 2. Calcium correction formulas

Formulas	Calculation	Units
Payne [a)]	total Ca − albumin + 4.0	total Ca: mg/dL<br>albumin: g/dL
Simplified [b)]	total Ca + 0.8 × (4.0 − albumin)	
James [c)]	total Ca + 0.48 × (3.99 − albumin)	
Orrell [d)]	total Ca + 0.71 × (3.4 − albumin)	
Berry [e)]	total Ca + 0.9 × (4.6 − albumin)	
Thode [f)]	total Ca × 2.7 / (1.7 + albumin/4.2)	
Antonio [g)]	$0.407 \times \text{total Ca}^{0.5} - 0.0337 \times \text{albumin}^{0.75} + 0.079$	

a) Br Med J 1973; 4: 643–646

b) Br Med J 1977; 1: 598

c) BMC Clin Pathol 2008; 8: 12

d) Clin Chim Acta 1971; 35: 483–489

e) Br Med J 1973; 4: 640–643

f) Scand J Clin Lab Invest 1989; 49: 217–223

g) Med Princ Pract 2016; 25: 219–226

Supplemental Table 3. Cross-classification between calcium measures and ionized calcium categories

Classification by total calcium, n	Classification by ionized calcium, n				Observed agreement
	Hypocalcemia	Normocalcemia	Hypercalcemia	Total	
Hypocalcemia	108	18	0	126	
Normocalcemia	172	342	3	517	70%
Hypercalcemia	0	1	3	4	
Total	280	361	6	647	

Classification by Payne formula, n	Classification by ionized calcium, n				Observed agreement
	Hypocalcemia	Normocalcemia	Hypercalcemia	Total	
Hypocalcemia	22	6	0	28	
Normocalcemia	257	339	2	598	56%
Hypercalcemia	1	16	4	21	
Total	280	361	6	647	

Classification by simplified formula, n	Classification by ionized calcium, n				Observed agreement
	Hypocalcemia	Normocalcemia	Hypercalcemia	Total	
Hypocalcemia	23	5	0	28	
Normocalcemia	257	349	2	608	58%
Hypercalcemia	0	7	4	11	
Total	280	361	6	647	

Classification by James formula, n	Classification by ionized calcium, n				Observed agreement
	Hypocalcemia	Normocalcemia	Hypercalcemia	Total	
Hypocalcemia	44	2	0	46	
Normocalcemia	236	357	2	595	63%
Hypercalcemia	0	2	4	6	
Total	280	361	6	647	

Classification by Orell formula, n	Classification by ionized calcium, n				Observed agreement
	Hypocalcemia	Normocalcemia	Hypercalcemia	Total	
Hypocalcemia	96	52	0	148	
Normocalcemia	184	308	1	493	63%
Hypercalcemia	0	4	2	6	
Total	280	364	3	647	

Classification by Berry formula, n	Classification by ionized calcium, n				Observed agreement
	Hypocalcemia	Normocalcemia	Hypercalcemia	Total	
Hypocalcemia	10	0	0	10	
Normocalcemia	256	317	0	573	52%
Hypercalcemia	14	44	6	64	
Total	280	361	6	647	

Classification by Thode formula, n	Classification by ionized calcium, n				Observed agreement
	Hypocalcemia	Normocalcemia	Hypercalcemia	Total	
Hypocalcemia	20	1	0	21	
Normocalcemia	258	338	2	598	56%
Hypercalcemia	2	22	4	28	
Total	280	361	6	647	

Classification by Antonio formula, n	Classification by ionized calcium, n				Observed agreement
	Hypocalcemia	Normocalcemia	Hypercalcemia	Total	
Hypocalcemia	49	2	0	51	
Normocalcemia	231	359	4	594	63%
Hypercalcemia	0	0	2	2	
Total	280	361	6	647	

Supplemental Table 4. Association between QT interval and ionized, total, and corrected calcium (RR interval additionally adjusted)

	standardized β (95% CI)	
	Unadjusted	Model 3
Ionized calcium	−0.02 (−0.10 to 0.06)	−0.12 (−0.16 to −0.07)
Total calcium	−0.03 (−0.11 to 0.04)	−0.06 (−0.11 to −0.01)
Corrected calcium		
Payne formula	−0.05 (−0.13 to 0.03)	−0.09 (−0.14 to −0.04)
Simplified formula	−0.05 (−0.13 to 0.03)	−0.09 (−0.14 to −0.04)
James formula	−0.05 (−0.13 to 0.03)	−0.08 (−0.13 to −0.03)
Orrell formula	−0.05 (−0.13 to 0.03)	−0.09 (−0.14 to −0.04)
Berry formula	−0.05 (−0.13 to 0.03)	−0.09 (−0.14 to −0.04)
Thode formula	−0.06 (−0.13 to 0.02)	−0.09 (−0.14 to −0.04)
Antonio formula	−0.04 (−0.12 to 0.03)	−0.07 (−0.12 to −0.02)

Model 3 was adjusted for age, sex, cardiac disease, QT-prolonging medications, estimated glomerular filtration rate, serum potassium, phosphate, magnesium levels, hypertension, and diabetes. β, regression coefficient; CI, confidence interval.

Supplemental Table 5. Association between QTc interval (Hodges formula) and ionized, total, and corrected calcium

	standardized β (95% CI)	
	Unadjusted	Model 3
Ionized calcium	−0.20 (−0.27 to −0.12)	−0.17 (−0.25 to −0.10)
Total calcium	−0.10 (−0.18 to −0.03)	−0.07 (−0.15 to 0.02)
Corrected calcium		
Payne formula	−0.07 (−0.15 to 0.00)	−0.13 (−0.21 to −0.05)
Simplified formula	−0.09 (−0.17 to −0.01)	−0.12 (−0.20 to −0.04)
James formula	−0.11 (−0.19 to −0.03)	−0.10 (−0.18 to −0.03)
Orrell formula	−0.10 (−0.17 to −0.02)	−0.12 (−0.20 to −0.04)
Berry formula	−0.08 (−0.16 to −0.00)	−0.13 (−0.20 to −0.05)
Thode formula	−0.09 (−0.17 to −0.02)	−0.13 (−0.20 to −0.05)
Antonio formula	−0.11 (−0.19 to −0.03)	−0.09 (−0.17 to −0.01)

Model 3 was adjusted for age, sex, cardiac disease, QT-prolonging medications, estimated glomerular filtration rate, serum potassium, phosphate, magnesium levels, hypertension, and diabetes. QTc, corrected QT; β, regression coefficient; CI, confidence interval.

Supplemental Table 6. Association between QTc prolongation (Hodges formula) and ionized, total, and corrected calcium

	Odds ratio (95% CI)	
	Unadjusted	Model 3
Ionized calcium	0.56 (0.44–0.70)	0.60 (0.47–0.77)
Total calcium	0.71 (0.58–0.88)	0.77 (0.61–0.97)
Corrected calcium		
Payne formula	0.77 (0.61–0.96)	0.72 (0.56–0.92)
Simplified formula	0.73 (0.58–0.90)	0.72 (0.56–0.90)
James formula	0.69 (0.55–0.86)	0.73 (0.57–0.91)
Orrell formula	0.71 (0.57–0.88)	0.72 (0.56–0.90)
Berry formula	0.75 (0.60–0.93)	0.72 (0.56–0.91)
Thode formula	0.72 (0.58–0.90)	0.71 (0.56–0.90)
Antonio formula	0.69 (0.56–0.85)	0.74 (0.59–0.92)

Odds ratio is expressed per 1-SD increase in each calcium measure.

Model 3 was adjusted for age, sex, cardiac disease, QT-prolonging medications, estimated glomerular filtration rate, serum potassium, phosphate, magnesium levels, hypertension, and diabetes. QTc, corrected QT; CI, confidence interval.

Supplemental Table 7. Association (R^2) with ionized calcium, classification agreement and weighted κ with hypoalbuminemia

	R^2	Classification agreement, %	κ (95% CI)
Total calcium	0.48	75	0.42 (0.24–0.59)
Payne formula	0.49	22	0.15 (0.09–0.22)
Simplified formula	0.53	29	0.10 (0.04–0.16)
James formula	0.54	41	0.11 (0.03–0.18)
Orrell formula	0.54	45	0.14 (0.05–0.24)
Berry formula	0.51	12	0.16 (0.10–0.22)
Thode formula	0.52	20	0.19 (0.12–0.25)
Antonio formula	0.53	50	0.18 (0.08–0.27)

Supplemental Table 8. Association between QTc interval and ionized, total, and corrected calcium in individuals with hypoalbuminemia

	standardized β (95% CI)	
	Unadjusted	Model 3
Ionized calcium	−0.23 (−0.41 to −0.05)	−0.25 (−0.44 to −0.06)
Total calcium	−0.08 (−0.27 to 0.10)	−0.10 (−0.30 to 0.10)
Corrected calcium		
Payne formula	−0.15 (−0.33 to −0.03)	−0.19 (−0.39 to 0.01)
Simplified formula	−0.14 (−0.32 to 0.04)	−0.18 (−0.38 to 0.02)
James formula	−0.12 (−0.30 to 0.06)	−0.15 (−0.35 to 0.05)
Orrell formula	−0.14 (−0.32 to 0.05)	−0.17 (−0.37 to 0.03)
Berry formula	−0.15 (−0.33 to 0.04)	−0.19 (−0.39 to 0.01)
Thode formula	−0.15 (−0.33 to 0.04)	−0.18 (−0.38 to 0.02)
Antonio formula	−0.11 (−0.29 to 0.08)	−0.13 (−0.33 to 0.07)

Model 3 was adjusted for age, sex, cardiac disease, QT-prolonging medications, estimated glomerular filtration rate, serum potassium, phosphate, magnesium levels, hypertension, and diabetes. QTc, corrected QT; β, regression coefficient; CI, confidence interval.

Supplemental Table 9. Association between QTc prolongation and ionized, total, and corrected calcium in individuals with hypoalbuminemia

	Odds ratio (95% CI)	
	Unadjusted	Model 3
Ionized calcium	0.53 (0.33–0.79)	0.51 (0.30–0.80)
Total calcium	0.80 (0.54–1.16)	0.83 (0.53–1.25)
Corrected calcium		
Payne formula	0.62 (0.41–0.91)	0.60 (0.37–0.92)
Simplified formula	0.64 (0.43–0.94)	0.62 (0.39–0.96)
James formula	0.70 (0.46–1.01)	0.69 (0.43–1.06)
Orrell formula	0.66 (0.43–0.96)	0.64 (0.40–0.99)
Berry formula	0.63 (0.42–0.92)	0.61 (0.38–0.94)
Thode formula	0.64 (0.42–0.93)	0.61 (0.38–0.95)
Antonio formula	0.74 (0.49–1.07)	0.74 (0.47–1.13)

Odds ratio is expressed per 1-SD increase in each calcium measure.
Model 3 was adjusted for age, sex, cardiac disease, QT-prolonging medications, estimated glomerular filtration rate, serum potassium, phosphate, magnesium levels, hypertension, and diabetes. QTc, corrected QT; CI, confidence interval.

Supplemental Table 10. Association between QTc interval and ionized, total, and corrected calcium in individuals stratified by alkalemia status

pH > 7.45	standardized β (95% CI)	
	Unadjusted	Model 3
Ionized calcium	-0.30 (-0.43 to -0.16)	-0.29 (-0.42 to -0.15)
Total calcium	-0.14 (-0.28 to -0.01)	-0.10 (-0.25 to 0.04)
Corrected calcium		
Payne formula	-0.12 (-0.26 to 0.01)	-0.17 (-0.31 to -0.04)
Simplified formula	-0.15 (-0.29 to -0.01)	-0.18 (-0.31 to -0.04)
James formula	-0.17 (-0.30 to -0.03)	-0.16 (-0.30 to -0.02)
Orrell formula	-0.16 (-0.30 to -0.02)	-0.17 (-0.31 to -0.04)
Berry formula	-0.14 (-0.28 to 0.00)	-0.18 (-0.31 to -0.04)
Thode formula	-0.14 (-0.28 to -0.01)	-0.17 (-0.31 to -0.04)
Antonio formula	-0.16 (-0.30 to -0.02)	-0.14 (-0.28 to 0.01)

Odds ratio is expressed per 1-SD increase in each calcium measure.
Model 3 was adjusted for age, sex, cardiac disease, QT-prolonging medications, estimated glomerular filtration rate, serum potassium, phosphate, magnesium levels, hypertension, and diabetes. QTc, corrected QT; CI, confidence interval.

pH ≤ 7.45	standardized β (95% CI)	
	Unadjusted	Model 3
Ionized calcium	-0.20 (-0.30 to -0.11)	-0.19 (-0.28 to -0.10)
Total calcium	-0.11 (-0.21 to -0.02)	-0.09 (-0.18 to 0.01)
Corrected calcium		
Payne formula	-0.09 (-0.19 to 0.00)	-0.13 (-0.23 to -0.03)
Simplified formula	-0.11 (-0.20 to -0.02)	-0.13 (-0.23 to -0.03)
James formula	-0.13 (-0.22 to -0.03)	-0.12 (-0.21 to -0.02)
Orrell formula	-0.12 (-0.21 to -0.02)	-0.13 (-0.23 to -0.03)
Berry formula	-0.10 (-0.20 to -0.01)	-0.13 (-0.23 to -0.03)
Thode formula	-0.11 (-0.20 to -0.02)	-0.13 (-0.23 to -0.03)
Antonio formula	-0.13 (-0.22 to -0.04)	-0.11 (-0.20 to -0.01)

Odds ratio is expressed per 1-SD increase in each calcium measure.
Model 3 was adjusted for age, sex, cardiac disease, QT-prolonging medications, estimated glomerular filtration rate, serum potassium, phosphate, magnesium levels, hypertension, and diabetes. QTc, corrected QT; CI, confidence interval.

Supplemental Table 11. Association between QTc prolongation and ionized, total, and corrected calcium in individuals stratified by alkalemia status

pH > 7.45	Odds ratio (95% CI)	
	Unadjusted	Model 3
Ionized calcium	0.42 (0.27–0.61)	0.48 (0.31–0.70)
Total calcium	0.77 (0.57–1.02)	0.81 (0.58–1.11)
Corrected calcium		
Payne formula	0.81 (0.60–1.07)	0.74 (0.53–1.02)
Simplified formula	0.76 (0.56–1.02)	0.73 (0.52–1.00)
James formula	0.73 (0.54–0.98)	0.74 (0.53–1.02)
Orrell formula	0.75 (0.55–1.00)	0.73 (0.52–1.00)
Berry formula	0.79 (0.58–1.04)	0.74 (0.52–1.01)
Thode formula	0.77 (0.57–1.03)	0.73 (0.52–1.00)
Antonio formula	0.74 (0.55–0.99)	0.77 (0.55–1.05)

Odds ratio is expressed per 1-SD increase in each calcium measure.
Model 3 was adjusted for age, sex, cardiac disease, QT-prolonging medications, estimated glomerular filtration rate, serum potassium, phosphate, magnesium levels, hypertension, and diabetes. QTc, corrected QT; CI, confidence interval.

pH ≤ 7.45	Odds ratio (95% CI)	
	Unadjusted	Model 3
Ionized calcium	0.65 (0.51–0.80)	0.67 (0.52–0.84)
Total calcium	0.78 (0.64–0.95)	0.84 (0.68–1.04)
Corrected calcium		
Payne formula	0.75 (0.60–0.91)	0.70 (0.55–0.89)
Simplified formula	0.72 (0.58–0.88)	0.71 (0.55–0.90)
James formula	0.72 (0.57–0.88)	0.76 (0.60–0.94)
Orrell formula	0.71 (0.57–0.88)	0.72 (0.56–0.90)
Berry formula	0.73 (0.59–0.90)	0.71 (0.55–0.89)
Thode formula	0.72 (0.57–0.88)	0.71 (0.55–0.90)
Antonio formula	0.74 (0.60–0.90)	0.79 (0.63–0.98)

Odds ratio is expressed per 1-SD increase in each calcium measure.
Model 3 was adjusted for age, sex, cardiac disease, QT-prolonging medications, estimated glomerular filtration rate, serum potassium, phosphate, magnesium levels, hypertension, and diabetes. QTc, corrected QT; CI, confidence interval.

Supplemental Figure 1. Scatter plot and correlation with ionized calcium

Scatter plots with fitted regression lines show the associations between ionized calcium and (a) total calcium, (b) Payne formula, (c) Simplified formula, (d) James formula, (e) Orrell formula, (f) Berry formula, (g) Thode formula, and (h) Antonio formula. Pearson's correlation coefficients (r) and coefficients of determination (R^2) are displayed in each panel.

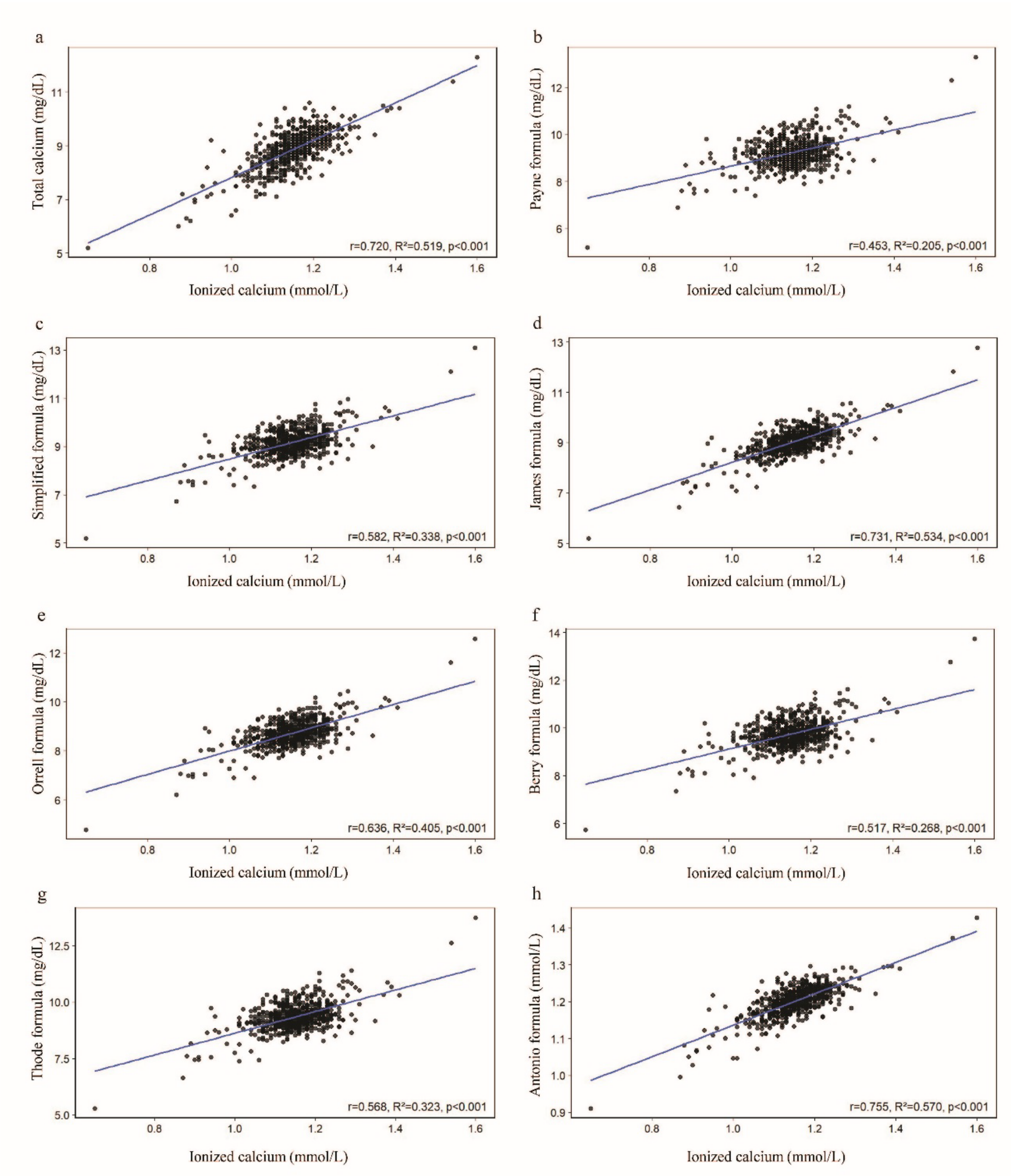

Supplemental Figure 2. Associations of calcium indices with QTc interval and QTc prolongation across clinical subgroups

Forest plots show standardized regression coefficients (β) and odds ratios (OR) for QTc (corrected QT) prolongation for each calcium measure, stratified by sex (male/female), age (<65 / ≥65 years), and kidney function (eGFR <60 / ≥60 mL/min/1.73 m²). All estimates were derived from Model 3. P values for interaction are displayed for both β and OR in each subgroup. Error bars represent 95% confidence intervals.

The number of participants in each subgroup was as follows: male (n=351), female (n=296); age <65 years (n=282), ≥65 years (n=365); <60 mL/min/1.73 m² (n=285), eGFR ≥60 mL/min/1.73 m² (n=362).

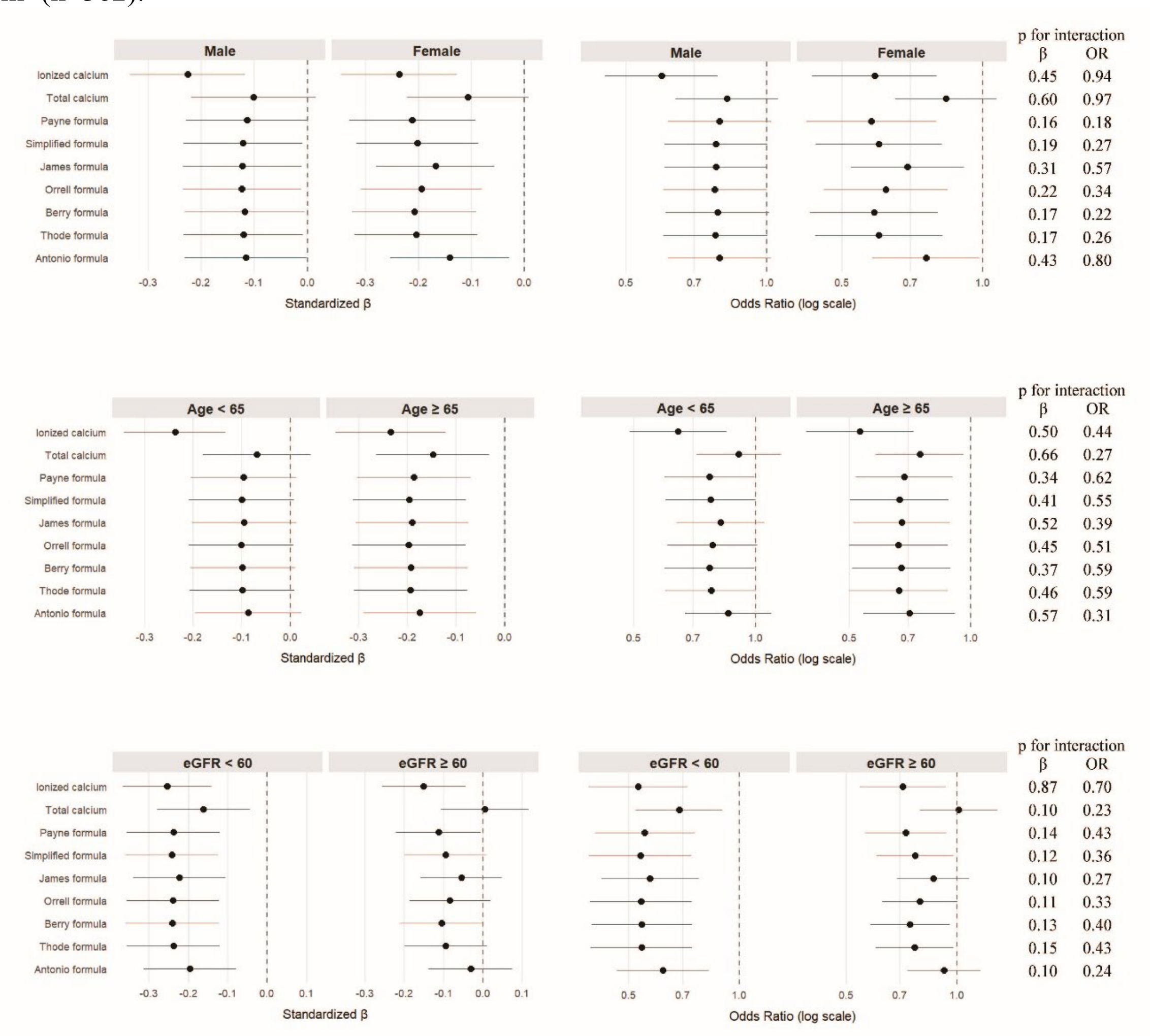

Supplement: Supplementary file 2 [file kidney360-7-1998-s002.pdf]
